# Dataset on the impact of implementing a shared governance model on the level of professional governance among nurses in Saudi Arabia: Insights from experimental data

**DOI:** 10.1016/j.dib.2024.110572

**Published:** 2024-05-31

**Authors:** Mahmoud Hamdan, Amar Hisham Jaaffar, Omar Khraisat

**Affiliations:** a King Saud Medical City, Riyadh, Saudi Arabia; b College of Graduate Studies (COGS), Universiti Tenaga Nasional (UNITEN), Putrajaya Campus, Jalan IKRAM-UNITEN, 43000 Kajang, Selangor; c Institute of Energy Policy and Research (IEPRe), Universiti Tenaga Nasional (UNITEN), Putrajaya Campus, Jalan IKRAM-UNITEN, 43000 Kajang, Selangor; d Faculty of Nursing, Al-Ahliyya Amman University, Amman, Jordan

**Keywords:** Shared governance, Professional nurse, Experimental, Saudi Arabia

## Abstract

Shared governance is a concept that has been gaining popularity in the nursing field. It is a framework that allows nurses to have a greater role in clinical decision-making. This approach recognizes the expertise and knowledge that nurses possess and allows them to be active participants in the decision-making process. It is a way to empower nurses and to ensure that the best possible care is being provided to patients. By promoting shared governance, nurses are able to work collaboratively with other healthcare professionals and provide high-quality care that is evidence-based and patient-centered. This article presents data that was collected in an empirical study to investigate the impact of implementing a shared governance model on the perceptions of professional governance among nurses working in a tertiary hospital in Saudi Arabia by measuring the level of shared governance from the lowest level, the traditional governance level (management and administration only), to the highest level, the self-governance level (staff only), through six dimensions of nursing professional governance, including personnel, information, resources, participation, practice, and goals. The study was conducted over 8 months between July 2022 to February 2023 with the involvement of a random sample of 200 clinical nurses who completed a structured questionnaire before and after the study interventions as part of quasi-research. The interventions included designing and implementing a shared governance model, and providing a shared governance training to clinical to nurse participants. The pretest-posttest experimental group showed that there were improvements in the level of shared governance (shared governance level - primarily management/administration with some staff input), which denotes the effectiveness of nursing professionals governance training among nurses working in a tertiary hospital in Saudi Arabia. The data used in this study can be utilized by future studies for benchmarking purposes.

Specifications Table

**Table:** 

Subject	Nursing and Health Professions
Specific subject area	Using a quasi-experimental research design to the outcome of shared governance.
Type of data	Table
How the data were acquired	Data were collected through a survey using a questionnaire. The tool was tested and used in previous literature.
Data Format	Raw and analyzed data
Description of data collection	The data were collected in a pretest-posttest study to investigate the impact of nursing shared governance model implementation on the level of professional governance among nurses. The study involved 200 frontline nurses through random sampling. The study used the Index of Professional Shared Governance (IPNG 3.0) questionnaire which includes 50 items divided into six shared governance dimensions using a five-point Likert scale. Eligibility criteria included nurses who are licensed and have at least six months experience in the current working unit. Each nurse received an invitation letter and signed a consent form. Data were collected before and after the study interventions which included designing and implementing a shared governance model including certain councils and processes, and providing shared governance training to nurse participants. Nurses’ responses were coded and analyzed using descriptive and inferential statistics. Descriptive statistic results showed the frequency and percentage of the responses for each question, while paired *t*-test showed the difference in professional governance dimensions before and after the interventions.
Data source location	City/Town/Region: Riyadh Country: Saudi Arabia
Data accessibility	Repository name: Mendeley data Data identification number: https://doi.org/10.17632/gpdjg8wpzb.5 Direct URL to data: https://data.mendeley.com/datasets/gpdjg8wpzb/5 See Reference [1]

## 1 Value of the Data

• The dataset can be used as a valuable baseline and benchmark for nursing and healthcare leaders who either plan to apply shared governance structures and processes in nursing or those who have already applied them as part of their journey to excellence.
• The data addresses the importance of measuring the level of professional governance among clinical nurses as it affects patient outcomes such as pressure injuries, patient falls, central line associated blood stream infections (CLABSI), catheter associated urinary tract infections (CAUTI), and patient satisfaction [[1], [2], [3]]. Since the perception of professional governance among nurses is correlated with these patient outcomes, measuring shared governance perceptions among nurses provides vital implications.
• Due to its advantages, this dataset is very pertinent to academics interested in undertaking descriptive and interventional studies aimed at enhancing nurse shared governance.
• The dataset offers an abundance of information on the effect of implementing a shared governance model in a practical clinical environment. Moreover, it can further identify the expected improvements in shared governance dimensions caused by implementing such interventions.
• The data can be utilized to replicate the study in different clinical settings or countries. This would also be helpful to compare the effectiveness of different improvement interventions.
• The dataset is intriguing because it enhances the paired-sample experiment for cross-sectional data as it has been collected based on two points of time (collected over eight months in 2022 and 2023).
• The data is also advantageous because it was collected using a stringent process. To find suitable tools to measure nursing professional governance perceptions, a thorough literature review was performed.
• The dataset is for an empirical study examining the effect of implementing a shared governance model of nursing professional governance. Only a few studies were conducted in this research type. The results showed significant improvement on professional governance dimensions resulted by the study interventions.

## 2 Objective

Professional governance involves a continuum of traditional governance, shared governance, and self-governance. Shared governance is a vital component of nursing practice, as it allows for collaboration and communication among nurses, management, and other healthcare professionals. By involving nurses in decision-making processes, shared governance promotes a sense of ownership and accountability in the workplace, which ultimately leads to improved patient outcomes and satisfaction. This approach also fosters a culture of innovation and continuous learning, as nurses are encouraged to share their ideas and perspectives. Overall, shared governance is essential for ensuring that nurses have a voice in shaping the future of healthcare and advancing the profession. The purpose of this survey was to examine the impact of implementing a shared governance model in improving the level of professional governance among clinical nurses. The dataset will add value to much research on shared governance structures and processes that aim to improve the level of professional governance among nurses.

## 3 Data Description

The dataset contains responses to the study questionnaire which was completed by the nurse participants. It includes the raw data of 200 clinical nurses including their sociodemographic characteristics. It also includes their perceptions of professional governance measured by 50 questions before and after the interventions. Participants were all licensed nurses with more than six months of experience in the current working unit. The questionnaire measures nurses’ perceptions of professional governance through six dimensions. Based on the overall scores, the level of professional governance is categorized into three levels; traditional governance where managers make most of the decisions, shared governance where decision are made by managers and staff, and self-governance where decisions are made mostly by the staff. Each item is the dataset is coded based on its position in the questionnaire. Sociodemographic characteristics including age, gender, marital status, level of education, years of experience, and working unit are shown in Table 1. Table 2 presents nurses’ perceptions of professional governance before and after designing and implementing a shared governance model, and providing nurses with a shared governance training. The table includes 50 professional governance items that are divided into six professional governance dimensions. In Table 3, the results of paired sample *t*-test shows that professional governance scores post interventions are significantly higher than professional governance scores before the interventions for the six dimensions as well as the overall professional governance scores.

**Table 1:** Socio-demographic information of participants (*N* = 200).

Variable	n (%)
**Age Categories**	
21–30 years	51 (25.5)
31–40 years	86 (43)
41–50 years	45 (22.5)
≥ 51 years	18 (9)
**Gender**	
Male	6 (3)
Female	194 (97)
**Level of Education**	
Diploma	18 (9)
Bachelor	157 (78.5)
Masters or above	25 (12.5)
**Marital status**	
Single	50 (25)
Married	125 (68.5)
Divorced	7 (3.5)
Widow	6 (3)
**Working experience**	
1–5 years	19 (9.5)
6–10 years	87 (43.5)
11–15 years	24 (12)
≥ 15 years	70 (35)
**Unit**	
Critical care	101 (50.5)
Non-critical care	99 (49.5)

**Table 2:** IPNG 3.0 results before and after intervention (*N* = 200).

Variable	Items	Pre-intervention					Post-intervention				
		Frequency, n (Percentage,%)					Frequency, n (Percentage,%)				
		Admin. only	Primarily admin. with some staff input	Equally shared	Primarily staff with some admin. input	Staff only	Admin. only	Primarily admin. with some staff input	Equally shared	Primarily staff with some admin. Input	Staff only
Personnel (PSL):											
PSL.1	Procedures for hiring and transferring nursing personnel	168 (84)	12 (6)	11 (5.5)	4 (2)	5 (2.5)	66 (33)	25 (12.5)	20 (10)	41 (20.5)	48 (24)
PSL.2	Written guidelines for disciplining nursing personnel	62 (31)	99 (49.5)	28 (14)	5 (2.5)	6 (3)	54 (27)	38 (19)	39 (19.5)	21 (10.5)	48 (24)
PSL.3	Creating new administrative or support positions	144 (72)	31 (15.5)	18 (9)	4 (2)	3 (1.5)	75 (37.5)	16 (8)	54 (27)	30 (15)	25 (12.5)
PSL.4	Creating new clinical positions	157 (77.5)	25 (12.5)	7 (3.5)	6 (3)	5 (2.5)	68 (34)	45 (22.5)	33 (16.5)	19 (9.5)	35 (17.5)
PSL.5	Policies regulating promotion of nursing personnel to management and leadership positions	144 (72)	31 (15.5)	12 (6)	6 (3)	7 (3.5)	78 (38)	29 (14.5)	8 (4)	37 (18.5)	48 (24)
PSL.6	Formulating annual unit budgets for personnel, supplies, equipment and education	116 (58)	53 (26.5)	18 (9)	8 (4)	5 (2.5)	75 (37.5)	45 (22.5)	35 (17.5)	19 (9.5)	26 (13)
PSL.7	Procedures for adjusting nursing salaries, raises and benefit	143 (71.5)	12 (6)	25 (12.5)	13 (6.5)	7 (3.5)	67 (33.5)	44 (22)	19 (9.5)	21 (10.5)	49 (24.5)
PSL.8	Organizational charts that show job titles and who reports to whom	126 (63)	55 (27.5)	8 (4)	6 (3)	5 (2.5)	67 (33.5)	36 (18)	48 ()24	20 (10)	29 (14.5)
PSL.9	Process for recommending and formulating annual unit budgets for personnel, supplies, major equipment and education	123 (61.5)	45 (22.5)	14 (7)	12 (6)	6 (6)	54 (27)	48 (24)	21 (10.5)	27 (13.5)	50 (25)
PSL.10	Recommending nursing salaries, raises and benefits	157 (77.5)	24 (12)	7 (3.5)	7 (3.5)	5 (2.5)	93 (46.5)	10 (5)	54 (27)	18 (9)	25 (12.5)
PSL.11	Conducting disciplinary action of nursing personnel	95 (47.5)	68 (34)	24 (12)	9 (4.5)	4 (2)	74 (37)	15 (7.5)	39 (19.5)	24 (12)	48 (24)
PSL.12	Mandatory RN credentialing levels (licensure, education, certifications) for hiring, continued employment, promotions and raises	162 (81)	12 (6)	14 (7)	7 (3.5)	5 (2.5)	63 (31.5)	45 (22.5)	20 (10)	31 (15.5)	41 (20.5)
Access to Information (ATI):											
ATI.1	Management's opinion of bedside nursing practice	44 (22)	70 (35)	60 (30)	13 (6.5)	13 (6.5)	18 (9)	25 (12.5)	41 (20.5)	55 (27.5)	61 (30.5)
ATI.2	Nurses’ satisfaction with their general practice	25 (12.5)	74 (37)	55 (27.5)	30 (15)	16 (8)	21 (10.5)	26 (13)	33 (16.5)	74 (37)	46 (23)
ATI.3	Nurses’ satisfaction with their salaries and benefits	93 (46.5)	51 (25.5)	36 (18)	12 (6)	8 (4)	33 (16.5)	28 (14)	47 (23.5)	49 (24.5)	43 (21.3)
ATI.4	Physician/nurse satisfaction with their collaborative practice	25 (12.5)	61 (30.5)	88 (44)	20 (10)	6 (3)	28 (14)	24 (12)	38 (19)	74 (37)	36 (18)
ATI.5	Current hospital status of nurse turnover and vacancies	108 (54)	49 (24.5)	24 (12)	14 (7)	5 (2.5)	44 (22)	27 (13.5)	34 (17)	57 (28.5)	38 (19)
ATI.6	Results of patient satisfaction surveys	33 (16.5)	36 (18)	101 (50.5)	17 (8.5)	13 (6.5)	28 (14)	23 (12.5)	48 (24)	45 (22.5)	56 (28)
ATI.7	requirements of surveying agencies (Joint Commission, state and federal government, professional groups)	81 (40.5)	43 (21.5)	63 (31.5)	8 (4)	5 (2.5)	23 (11.5)	28 (14)	87 (43.5)	28 (14)	34 (17)
ATI.8	Hospital's strategic plans for the next few years	93 (46.5)	48 (24)	46 (23)	9 (4.5)	4 (2)	48 (24)	47 (23.5)	46 (23)	27 (13.5)	32 (16)
ATI.9	Unit and nursing departmental goals and objectives for this year	43 (21.5)	68 (34)	63 (31.5)	20 (10)	6 (3)	55 (27.5)	15 (7.5)	47 (23.5)	26 (13)	57 (28.5)
Influence Over Resources (IOR):											
IOR.1	Consulting hospital services outside of nursing (e.g., dietary, social service, pharmacy, human resources, finance)	88 (44)	31 (15.5)	62 (31)	19 (9.5)	0 (0)	36 (18)	42 (21)	77 (37.5)	20 (10)	25 (12.5)
IOR.2	Regulating the flow of patient admissions, transfers, and discharges	32 (16)	75 (37.5)	74 (37)	13 (6.5)	6 (3)	37 (18.5)	19 (9.5)	56 (28)	32 (16)	56 (28)
IOR.3	Procedures for controlling the flow of patient admissions, transfers and discharges	51 (25.5)	81 (40.5)	36 (18)	19 (9.5)	13 (6.5)	32 (16)	65 (32.5)	17 (8.5)	32 (31)	54 (27)
IOR.4	Formal mechanisms for consulting and enlisting the support of hospital service outside of nursing (e.g., dietary, social service, pharmacy, physical therapy)	69 (34.5)	69 (34.5)	37 (18.5)	25 (12.5)	0 (0)	48 (24)	53 (26.5)	27 (13.5)	23 (11.5)	49 (24.5)
IOR.5	Daily methods for monitoring and obtaining supplies for nursing care and support functions	20 (10)	98 (48)	38 (19)	32 (16)	12 (6)	42 (21)	36 (18)	27 (13.5)	33 (16.5)	62 (31)
IOR.6	Consulting nursing services outside of the unit (e.g., administration, psychiatric, medical-surgical)	88 (44)	62 (31)	19 (9.5)	19 (9.5)	12 (6)	37 (18.5)	20 (10)	72 (36)	36 (18)	35 (17.5)
IOR.7	Formal mechanisms for consulting and enlisting the support of nursing services outside of the unit (e.g., administration, psychiatric, medical-surgical)	88 (44)	62 (31)	25 (12.5)	19 (9.5)	6 (3)	53 (26.5)	49 (24.5)	25 (12.5)	24 (12)	49 (24.5)
IOR.8	Procedures for determining daily patient care assignments	28 (14)	37 (18.5)	48 (24)	49 (29.5)	38 (19)	24 (12)	30 (15)	18 (9)	52 (26)	76 (38)
IOR.9	Making daily patient care assignments for nursing personnel	13 (6.5)	40 (20)	52 (26)	50 (25)	45 (22.5)	9 (4.5)	19 (9.5)	64 (32)	51 (25.5)	57 (28.5)
Participation in Committee Structures (PIC):											
PIC.1	Forming new unit committees	69 (34.5)	49 (24.5)	63 (31.5)	12 (6)	7 (3.5)	53 (26.5)	53 (26.5)	25 (12.5)	28 (14)	41 (20.5)
PIC.2	Forming new nursing departmental committees	81 (40.5)	56 (28)	50 (25)	8 (4)	5 (2.5)	31 (15.5)	39 (19.5)	73 (36.5)	29 (14.5)	28 (14)
PIC.3	Participation in multidisciplinary professional committees (physicians, other hospital professions and departments) for collaborative practice	70 (35)	69 (34.5)	36 (18)	18 (9)	7 (3.5)	33 (16.5)	62 (31)	42 (21)	21 (10.5)	42 (24)
PIC.4	Forming new hospital administration committees	156 (78)	25 (12.5)	12 (6)	7 (3.5)	0 (0)	47 (23.5)	65 (32.5)	35 (17.5)	5 (2.5)	48 (24)
PIC.5	Participation in unit committees for administrative matters such as staffing, scheduling and budgeting	93 (46.5)	55 (27.5)	39 (19.5)	10 (5)	3 (1.5)	29 (24.5)	43 (21.5)	30 (15)	57 (27.5)	41 (20.5)
PIC.6	Forming new multidisciplinary professional committees	113 (56.5)	42 (21)	32 (16)	11 (5.5)	2 (1)	43 (21.5)	61 (30.5)	33 (16.5)	20 (10)	43 (21.5)
PIC.7	Participation in nursing departmental committees for administrative matters such as staffing, scheduling, and budgeting	95 (47.5)	73 (36.5)	19 (9.5)	9 (4.5)	4 (2)	26 (13)	42 (21)	32 (16)	58 (29)	42 (21)
PIC.8	Participation in hospital administration committees for matters such as employee benefits and strategic planning	125 (67.5)	37 (18.5)	31 (15.5)	7 (3.5)	0 (0)	38 (19)	47 (23.5)	56 (28)	37 (18.5)	22 (11)
Control Over Professional Practice (COP):											
COP.1	Determining what activities nurses can do at bedside	19 (9.5)	68 (34)	70 (35)	30 (15)	13 (6.5)	12 (6)	26 (13)	47 (23.5)	49 (24.5)	66 (33)
COP.2	Developing and evaluating patient care standards and quality assurance/improvement activities	25 (12.5)	87 (43.5)	51 (25.5)	30 (15)	7 (3.5)	10 (5)	21 (10.5)	90 (45)	48 (24)	31 (15.5)
COP.3	Assessing and providing for the professional/educational development of the nursing staff	6 (3)	101 (50.5)	62 (31)	26 (13)	5 (2.5)	32 (16)	32 (16)	59 (29.5)	49 (24.5)	28 (14)
COP.4	Determining methods of nursing care delivery (e.g., primary, team, case management)	38 (19)	63 (31.5)	74 (37)	21 (10.5)	4 (2)	34 (17)	37 (18.5)	45 (22.5)	43 (21.5)	41 (20.5)
COP.5	Determining activities of ancillary nursing personnel (aides, unit clerks, etc.)	62 (31)	70 (35)	55 (27.5)	7 (3.5)	6 (3)	59 (29.5)	45 (22.5)	28 (14)	25 (22.5)	43 (21.5)
COP.6	Setting levels of qualifications for nursing positions	125 (62.5)	44 (22)	12 (6)	10 (5)	9 (4.5)	62 (31)	13 (6.5)	63 (31.5)	30 (15)	32 (16)
COP.7	Selecting products used in nursing care	95 (47.5)	68 (34)	24 (12)	7 (3.5)	6 (3)	24 (12)	28 (14)	48 (24)	59 (29.5)	41 (20.5)
Goal Setting and Conflict Resolution (GSR):											
GSR.1	Negotiate solutions to conflicts between professional nurses and physicians	50 (25)	70 (35)	61 (30.5)	12 (6)	7 (3.5)	41 (20.5)	65 (32.5)	26 (13)	37 (18.5)	31 (15.5)
GSR.2	Negotiate solutions to conflicts between professional nurses and other hospital services (respiratory, dietary, etc.)	45 (22.5)	81 (40.5)	67 (33.5)	5 (2.5)	2 (1)	39 (19.5)	38 (19)	41 (20.5)	51 (25.5)	31 (15.5)
GSR.3	Negotiate solutions to conflicts between professional nurses and nursing management	24 (12)	108 (54)	61 (30.5)	4 (2)	3 (1.5)	34 (17)	64 (32)	22 (11)	31 (15.5)	49 (24.5)
GSR.4	Negotiate solutions to conflicts between professional nurses and hospital administration	62 (31)	94 (47)	37 (18.5)	7 (3.5)	0 (0)	46 (23)	57 (28.5)	34 (17)	37 (18.5)	26 (13)
GSR.5	Negotiate solutions to conflicts among professional nurses	37 (18.5)	69 (34.5)	69 (34.5)	12 (6)	13 (6.5)	38 (19)	51 (25.5)	24 (12)	36 (18)	51 (25.5)

**Table 3:** Comparing shared governance scores before and after interventions using paired samples *t*-test (*N* = 200).

Dimensions	Mean	Std. Deviation	t	Sig
PSL- Pre	18.760	8.674	−10.274	.000
PSL- Post	31.942	15.399		
ATI- Pre	20.525	7.126	−9.442	.000
ATI- Post	29.010	10.499		
IOR- Pre	22.000	7.252	−8.132	.000
IOR- Post	28.300	8.709		
PIC- Pre	14.640	6.092	−10.687	.000
PIC- Post	23.285	9.558		
COP- Pre	15.950	5.228	−10.021	.000
COP- Post	21.995	6.916		
GSR- Pre	11.160	3.932	−6.319	.000
GSR- Post	14.485	6.423		
Total Shared Governance- Pre	103.035	30.155	−11.212	.000
Total Shared Governance- Post	149.017	49.731		

*Note:* Traditional Governance Score (Management/Administration Only) : 50–100; Shared Governance (1Note: 1) Traditional Governance Score (Management/Administration Only) : 50–100; 2) Shared Governance (Primarily management/administration with some staff input): 101 – 149; 3) Shared Governance (Equally shared by staff and management/administration): 150; 4) Shared Governance (Primarily staff with some management/administration): 151 – 200; and 5) Self Governance (Staff only) : 201 – 250 [7].

## 4 Experimental Design, Materials and Methods

### 4.1 Participants, response rate and data collection procedure

A cross-sectional study was conducted from July 2022 to February 2023 to examine the impact of implementing a shared governance model on the level of professional governance among clinical nurses working in a tertiary hospital in Riyadh. The unit of analysis for this study is the individual. The random sampling technique was used to reduce selection bias and ensure that each stratum of the study population was accurately presented. A random sample of 240 nurses was invited to participate in the study, but 200 nurses only completed the self-administered questionnaire with an acceptable response rate of 83 % [1,2]. The total population of clinical nurses working in the hospital is 2200 nurses. The sample size was determined based on a power primer [3,4]. G*Power analysis suggested that a total of 146 participants were required to attain a statistical power of 80 %.

### 4.2 Data Collection and instruments

Ethical approval was obtained prior to the study from the Institutional Review Board at the study hospital. Consent was taken from each participant to ensure that participants’ anonymity and confidentiality are maintained. Data was collected using a questionnaire that consists of two parts; demographic data and the Index of Professional Nursing Governance (IPNG 3.0) (add reference). Demographic data included the age, gender, marital status, level of education, years of experience, and working unit for nurse participants. The language used in the questionnaire is English since English is the first language in Saudi hospitals, including study hospitals. Therefore, the language translation process is not required. Non-response bias, collinearity, and outliers were not serious issues for this study as shown in [1]. The questionnaire was tested through a pilot study with a thirty number of respondents to check the clarity of the questionnaire in terms of content and wording. Based on this pilot test, amendments were made before performing the actual survey.

The IPNG is a five-point Likert scale self-administered questionnaire consisting of six dimensions and 50 items to measure the level of professional governance among nurses [1,5]. Professional governance dimensions, including personnel, access to information, influence over resources, participation in committee structures, control over professional practice, goal setting, and conflict resolution, have shown good reliability value, as reflected by the value of Cronbach's α as shown in Table 4. The five-point Likert scale options include “management/ administration only”, “primary management/ administration with some staff input”, “equally shared between staff and management/ administration”, “primary staff with some management/ administration input”, and “staff only”. Based on the total scores, the level of professional governance can be categorized as traditional governance, shared governance, or self-governance. Shared governance zone is characterized by decision making process that is made by both of managers and staff [6,7]. The IPNG is the only valid and reliable tool for measuring shared governance [6]. Descriptive statistics and paired *t*-test were performed using SPSS statistics version 25.

**Table 4:** Internal consistency of items for professional governance.

Items	Cronbach's alpha	Number of Items
Personnel	0.9340	12
access to information	0.9030	9
influence over resources	0.8820	9
participation in committee structures	0.9090	8
control over professional practice	0.8720	7
goal setting and conflict resolution	0.9190	5

Data was collected before and after the study interventions. These interventions included designing and implementing a shared governance model among nurses. Shared governance is usually applied in the hospitals for its positive impact on nursing and patient outcomes, and as part of the nurses’ journey toward excellence [[8], [9], [10], [11], [12], [13]]. This shared governance model included a number of main shared governance councils; leadership council, practice council, quality and patient safety council, education and professional development council, recruitment and retention council, research and evidence-based practice council, and unit-based council (UBC) chairpersons council. In addition, the model included a unit-based council (UBC) in each nursing unit. The main shared governance councils included members from clinical nurses and were led by nursing leaders, while the UBCs involved members from clinical nurses and were led by clinical nurses as well. UBCs meet monthly to discuss clinical issues related to their clinical unit and submit ideas for improvement to the main shared governance councils in case they have opportunities for improvement. The flow of the ideas of improvement was designed in a simple chart for nurses, and the roles and responsibilities of the shared governance councils and the UBCs were clarified in the shared governance bylaws and the council charters.

## Ethics Statement

The data gathering procedure complied with the Declaration of Helsinkiʼs ethical standards. Anonymity was maintained when questionnaire was used and when the gathered data was coded. A brief summary of the study's scope and goals and an online permission form were supplied on the first page of the questionnaire. Access to the questionnaire was made available to survey respondents who decided to participate. Participation was voluntary and no nurse was forced to participate in the survey. The reference number is H1R1–27-Jul22–03.

## CRediT authorship contribution statement

**Mahmoud Hamdan:** Conceptualization, Methodology, Data curation, Investigation, Writing – review & editing. **Amar Hisham Jaaffar:** Data curation, Validation, Writing – review & editing. **Omar Khraisat:** .

## Data Availability

Dataset on The Impact of Implementing a Shared Governance Model on The Level of Professional Governance among Nurses in Saudi Arabia: Insights from Experimental Data (Original data) (Mendeley Data).

## Appendix Supplementary materials

Image, application 1

Image, application 2

## References

[1] Jaaffar A.H., Hamdan M. (2023). Dataset on the impact of implementing a shared governance model on the level of professional governance among nurses in Saudi Arabia: insights from experimental data. Mendeley Data.

[2] Weaver S.H., Hess R.G., Williams B., Guinta L., Paliwal M. (2018). Measuring shared governance: one healthcare system's experience. Nurs. Manag..

[3] Kanninen T.H., Häggman-Laitila A., Tervo-Heikkinen T., Kvist T. (2019). Nursing shared governance at hospitals – it's Finnish future?. Leadersh. Health Serv..

[4] Cohen J. (1992). A power primer. Psychol. Bull..

[5] Cohen J. (1988).

[6] Hess R. (1998). Measuring nursing governance. Nurs. Res..

[7] Hess R. (2020). Shared governance is everywhere!. Insight: J. Am. Soc. Ophthalm. Regist. Nurs..

[8] Hess R. (2004). From bedside to boardroom – nursing shared governance. Online J. Issues Nurs..

[9] Mahfouz H.H.E., Ebraheem S.M.A., Mahdy A.Y. (2019). The relation among shared governance, empowerment and job involvement as perceived by medical-surgical nursing staff. Evid. Nurs. Res..

[10] Ayaad O., Alloubani A., Thiab F., Yousef D., Banat B. (2018). Adopting a shared governance model to improve nurses’ working environments. Br. J. Healthc. Manag..

[11] Kutney-Lee A., Germack H., Hatfield L., Kelly S., Maguire P., Dierkes A., Del Guidice M., Aiken L.H. (2016). Nurse engagement in shared governance and patient and nurse outcomes. J. Nurs. Admin..

[12] Speroni K.G., Wisner K., Stafford A., Haines F., Al-Ruzzieh M.A., Walters C., Budhathoki C. (2021). Effect of shared governance on nurse-sensitive indicator and satisfaction outcomes: an international comparison. J. Nurs. Admin..

[13] Speroni K.G., Wisner K., Ober M., Haines F., Walters C., Budhathoki C. (2021). Effect of shared governance on nurse-sensitive indicator and satisfaction outcomes by magnet® recognition status. J. Nurs. Adm..

