# Supplementary material for: Dataset on the impact of implementing a shared governance model on the level of professional governance among nurses in Saudi Arabia: Insights from experimental data
Source: Data Brief. 2024 May 31;55:110572. doi: 10.1016/j.dib.2024.110572 (PMC11222808; doi:10.1016/j.dib.2024.110572)
Supplement: Supplementary file 1 [file mmc1.pdf]

Kingdom of Saudi Arabia
Ministry of Health
King Saud Medical City

مدينة الملك سعود الطبية
KING SAUD MEDICAL CITY

المـملكة العــربية الســعوديـــة
وزارة الــصحة
مدينة الملك سـعود الطبية

IRB Registration Number with KACST, KSA: H-01-R-053
IRB Registration Number U.S. Department of HHS IORG #: IORG0010374

# - Memorandum -

Date: August 29, 2022

Proposal Reference No.	: **H1RI-27-Jul22-03**
Proposal Title	: "The Effect of Transformational Leadership and Shared Governance on Patient Safety Outcomes from the Perspective of Nurses in Saudi Arabia: The Mediating Role of Patient Safety Culture"
PI	: Mr. Mahmoud Azmi Ahmad Hamdan
Co-Investigators	: None
Type of Review	: Modification
Category of Approval	: Exempt
Date of IRB Approval-Expiration (Validity)	: 29/08/2022 28/08/2023 (12 months)

Dear Mr. Mahmoud Azmi Ahmad Hamdan,

We are pleased to inform you that the above-referenced research proposal has been reviewed and was approved. The Institutional Review Board (IRB) committee found that the research met the applicability criteria and was eligible for exempt review. However, to commence the collection of data a permission letter must be issued from the Director of the Research Center first.

This approval is valid for **12 months** from the date of IRB review when approval is granted. The approval will no longer be in effect on the date listed above as the IRB expiration date. Please note that you are obligated to submit the following to IRB committee:

1. progress/final report on the **12 months (28-Aug-2023)** (or earlier in the case the study has completed)
2. any manuscript resulting from this research for approval by IRB before submission to journals for publication.

The approval of the conduct of this proposal will be automatically suspended after 12 months, in the case the Progress Report (or Final Report, if relevant) is pending acceptance. You also need to notify the Research Centre as soon as possible in case of:

1. any amendments to the proposal;
2. termination of the study;
3. any serious or unexpected adverse events;
4. any event or new information that may affect the benefit/risk ratio of the proposal.

All records relating to the research including consent form must be retained and available for audit for at least 3 years after the research has ended.

We wish you every success in your research endeavors.

Dr. Faisal Almazroua
Chairman, Institutional Review Board (IRB)
King Saud Medical City Riyadh, KSA

وزارة الصحة المملكة العربية السعودية
مدينة الملك سعود الطبية
King Saud Medical City
لجنة أخلاقيات البحوث (IRB)

☎435-5555 Ext. 2345 irb@ksmc.med.sa
