# Supplementary material for: Dataset on the impact of implementing a shared governance model on the level of professional governance among nurses in Saudi Arabia: Insights from experimental data
Source: Data Brief. 2024 May 31;55:110572. doi: 10.1016/j.dib.2024.110572 (PMC11222808; doi:10.1016/j.dib.2024.110572)
Supplement: Supplementary file 2 [file mmc2.pdf]

Journal Pre-proof

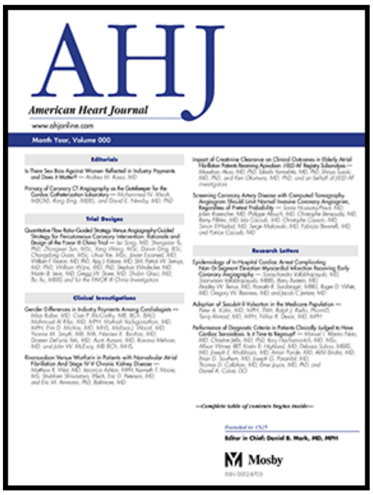

Seattle Proportional Risk Model in GISSI-HF: Estimated Benefit of ICD in Patients with EF less than 50%

Lee B. Bockus MD PhD , Ramin Shadman MD , Jeanne E. Poole MD , Todd F. Dardas MD , Donata Lucci MS , Jennifer Meessen MSc , Roberto Latini MD , Aldo Maggioni MD , Wayne C. Levy MD

PII: S0002-8703(24)00129-7
DOI: https://doi.org/10.1016/j.ahj.2024.05.014
Reference: YMHJ 6970

To appear in: *American Heart Journal*

Received date: February 22, 2024
Accepted date: May 25, 2024

Please cite this article as: Lee B. Bockus MD PhD , Ramin Shadman MD , Jeanne E. Poole MD , Todd F. Dardas MD , Donata Lucci MS , Jennifer Meessen MSc , Roberto Latini MD , Aldo Maggioni MD , Wayne C. Levy MD , Seattle Proportional Risk Model in GISSI-HF: Estimated Benefit of ICD in Patients with EF less than 50%, *American Heart Journal* (2024), doi: https://doi.org/10.1016/j.ahj.2024.05.014

This is a PDF file of an article that has undergone enhancements after acceptance, such as the addition of a cover page and metadata, and formatting for readability, but it is not yet the definitive version of record. This version will undergo additional copyediting, typesetting and review before it is published in its final form, but we are providing this version to give early visibility of the article. Please note that, during the production process, errors may be discovered which could affect the content, and all legal disclaimers that apply to the journal pertain.

© 2024 Published by Elsevier Inc.

Journal Pre-proof

Highlights

- Primary prevention ICD guidelines include EF ≤35% and NYHA class II to IV
- The multivariable SPRM predicts ICD benefit across both LVEF ≤ 35% and 36%-50%
- SPRM predicts benefit in LVEF up to 50% in cases of high proportional risk of SCD
- The concept of proportional risk should be incorporated into ICD guidelines

Journal Pre-proof

Journal Pre-proof

Seattle Proportional Risk Model in GISSI-HF: Estimated Benefit of ICD in Patients with EF less than 50%

Brief title: Estimated Benefit of ICD in EF <50%

Lee B. Bockus MD PhD,[a] Ramin Shadman MD,[b] Jeanne E. Poole MD,[a] Todd F. Dardas MD,[a] Donata Lucci MS,[c] Jennifer Meessen MSc,[d] Roberto Latini MD,[d] Aldo Maggioni MD,[d] Wayne C. Levy MD[a]

Word count: 2400

From the [a]University of Washington, Department of Medicine, Seattle, WA; [b]Southern California Permanente Medical Group, Los Angeles, CA; [c]Associazione Nazionale Medici Cardiologi Ospedalieri (ANMCO), Florence, Italy; [d]Institute for Pharmacological Research Mario Negri IRCCS, Department of Acute Brain and Cardiovascular Injury, Milano, Italy

Funding for this analysis: None

Disclosures: Dr Poole has research contracts direct to the University of Washington from Biotronik, Boston Scientific, Medtronic, Abbott, and Kestra. Dr Maggioni has served as a consultant to Novartis, Bayer, and AstraZeneca outside the present work. Dr Levy serves on the Clinical Endpoint Committee, CardioMems (Abbott), SOLVE-CRT (EBR Systems Inc.), ANTHEM-HF (Liva Nova), the Steering Committee for rēST (Respircardia), EMPOWER (Cardiac Dimensions), and Consultant for Medtronic, Impulse Dynamics and Kestra Medical, and has received research funding from the Medtronic External Research Program. The rest of the authors have no relationships relevant to the contents of this paper to disclose.

Address for Correspondence
Wayne C. Levy, Division of Cardiology, University of Washington, Box 3564222, 1959 Northeast Pacific Street, Seattle WA 98177. E-mail: levywc@uw.edu.

Twitter: @WCL92689433
The Seattle Proportional Risk Model (SPRM) predicts that only half of patients in the GISSI-HF registry that meet current guidelines derive a meaningful benefit from ICD implantation, and that 43% of patients with an ejection fraction ranging from 36% to 50% would benefit from an ICD. #SPRM #SHFM #SCD

Abstract

Background The Seattle Proportional Risk Model (SPRM) estimates the proportion of sudden cardiac death (SCD) in heart failure (HF) patients, identifying those most likely to benefit from implantable cardioverter-defibrillator (ICD) therapy (those with ≥50% estimated proportion of SCD). The GISSI-HF trial tested fish oil and rosuvastatin in HF patients. We used the SPRM to

Journal Pre-proof

evaluate its accuracy in this cohort in predicting potential ICD benefit in patients with EF ≤50% and an SPRM-predicted proportion of SCD either ≥50% or <50%.

Methods The SPRM was estimated in patients with EF ≤50% and in a logistic regression model comparing SCD with non-SCD.

Results We evaluated 6,750 patients with EF ≤50%. There were 1,892 all-cause deaths, including 610 SCDs. Fifty percent of EF ≤35% patients and 43% with EF 36% to 50% had an SPRM of ≥50%. The SPRM (OR: 1.92, $P < 0.0001$) accurately predicted the risk of SCD vs non-SCD with an estimated proportion of SCD of 44% vs the observed proportion of 41% at 1 year.

By traditional criteria for ICD implantation (EF ≤35%, NYHA class II or III), 64.5% of GISSI-HF patients would be eligible, with an estimated ICD benefit of 0.81. By SPRM >50%, 47.8% may be eligible, including 30.2% with EF >35%. GISSI-HF participants with EF ≤35% with SPRM ≥50% had an estimated ICD HR of 0.64, comparable to patients with EF 36% to 50% with SPRM ≥50% (HR: 0.65).

Conclusions The SPRM discriminated SCD vs non-SCD in GISSI-HF, both in patients with EF ≤35% and with EF 36% to 50%. The comparable estimated ICD benefit in patients with EF ≤35% and EF 36% to 50% supports the use of a proportional risk model for shared decision making with patients being considered for primary prevention ICD therapy.

Keywords: implantable cardioverter-defibrillator, sudden cardiac death, risk stratification

Introduction

The use of an implantable cardioverter-defibrillator (ICD) for primary prevention is recommended in patients with heart failure (HF) and reduced ejection fraction (EF), irrespective of etiology and comorbidities.[1-3] In patients with ischemic HF, the absolute risks of all-cause and sudden cardiac death (SCD) are higher, and the evidence for ICD benefit is stronger, than in patients with non-ischemic HF.[4-7] The primary benefit of an ICD is its ability to terminate potentially life threatening ventricular tachyarrhythmias. Hence, the benefit is greatest in patients most likely to die of SCD v. non-SCD causes. Multiple factors influence the proportional risk of SCD such as, age, comorbidities, and functional HF class. Moving beyond LVEF as the primary determinant of ICD decision making may result in a more equitable and effective use of ICD therapy.

Journal Pre-proof

The Seattle Proportional Risk Model (SPRM) has been shown to estimate the proportion of sudden cardiac death (SCD) in heart failure (HF) patients.[8] The greatest ICD benefit has been shown in those with ≥50% estimated proportion of SCD in the SCD-HeFT,[9] DANISH,[10] and HF-ACTION[11] trials and in the NCDR ICD Registry.[12] For example, in the SCD-HeFT analysis, the presence of an ICD in patients with SPRM ≥50% was associated with a 76% reduction in SCD and 44% reduction in all-cause mortality. Conversely, there was no ICD benefit for SCD or all-cause mortality in the quartile with SPRM <50% and markedly higher non-SCD rates. This is compared to the overall 27% mortality reduction of an ICD in the SCD-HeFT analysis based upon LVEF ≤35% and NYHA Class II-III.

To expand on these previous analyses, in this report we have utilized the GISSI-HF trial data set to study patients with higher ejection fractions (up to 50%) as well as the study of comorbidities that were not well represented in other cohorts. In the present analysis of the GISSI-HF trial, we applied combinations of previously validated SCD and HF risk prediction models to identify patients at greater risk of SCD who would be more likely to benefit from an ICD. The models used were the Seattle Proportional Risk Model (SPRM), which estimates the proportional risk of sudden vs nonsudden cardiac death,[8] and the Seattle Heart Failure Model (SHFM),[13] which estimates overall mortality.

Methods

Patient selection. GISSI-HF was a randomized, double-blind, placebo-controlled, multicenter trial testing fish oil and rosuvastatin in a large population of patients with symptomatic heart

Journal Pre-proof

failure of any cause.[14] Entry criteria included chronic heart failure of New York Heart Association (NYHA) class II to IV, irrespective of cause and left ventricular EF, with random assignment of 6,975 patients to n-3 polyunsaturated fatty acids or placebo. Patients were followed up for a median of 3.9 years, with the primary end points of time to death and time to death or admission to hospital for cardiovascular reasons. Amongst the overall population, there were 6,750 patients who had an ejection fraction ≤50% and were included in this analysis.

Risk stratification and outcomes. The SPRM (Seattle Proportional Risk Model) is a multinomial logistic regression multivariable model used to estimate the proportion of SCD vs other causes of mortality (non-SCD).[8] Advancing age, diabetes mellitus, hyponatremia, and chronic kidney disease all decrease the proportion of SCD, while male sex, NYHA class II (vs III or IV), lower EF, digoxin use, systolic blood pressure closer to 140 mm Hg, and higher BMI increase the proportion of SCD, based on the previously described risk model (interactive model is available at https://depts.washington.edu/sprm/).[8] The SHFM (Seattle Heart Failure Model - Cox proportional hazards model) was calculated as previously described to estimate annual mortality.[13] Lymphocytes (percentages, unavailable in GISSI-HF) were extrapolated using a comparable population based on age, systolic blood pressure, EF, WBC, hemoglobin, total cholesterol, and digoxin.[15] Clinical outcomes, focused on SCD, were adjudicated by a clinical event committee. Based on the study design, GISSI-HF allowed us to test whether additional comorbidities when added to the SPRM improved the mode-of-death prediction in a multinomial logistic regression model of SCD vs non-SCD. Variables evaluated for potential addition to SPRM were ICD (present prior to death), stroke, ischemic etiology, coronary artery bypass graft surgery (CABG), percutaneous coronary intervention (PCI), chronic obstructive pulmonary

Journal Pre-proof

disease (COPD), history of cancer, peripheral artery disease, atrial fibrillation, QRS duration on electrocardiogram, and the use of angiotensin-converting enzyme inhibitors, angiotensin receptor blockers, mineralocorticoid receptor antagonists, and beta blockers.

Statistical analyses. Baseline differences between patients according to median SPRM and SHFM scores were tested with Kruskal-Wallis. We derived Kaplan-Meier curves (Placebo group for SCD-HeFT, GISSI-HF EF ≤35% and GISSI-HF EF 36-50%) for all-cause mortality, SCD, and non-SCD, with censoring as alive for competing risk of death. SCD and non-SCD 5-year rates were estimated by multiplying the baseline SPRM percentage of SCD by the 5-year SHFM-estimated all-cause mortality. The graphic data of 5-year SCD vs. non SCD were plotted using the quartiles groupings of the SPRM model within SCD-HeFT and the ICD benefit observed for the SPRM quartile within SCD-HeFT. The ICD benefit for all-cause mortality varied markedly within SCD-HeFT. For example, the observed ICD benefit for SPRM 40%, 50%, 60%, and 70% was 0.95, 0.76. 0.63, and 0.50 respectively. The predicted ICD benefit is estimated for every patient within GISSI-HF limiting the ICD hazard ratio to ≤1.0 (i.e. no ICD benefit). SCD-HeFT patient level data were utilized to illustrate the ICD benefit by SPRM quartile grouping as previously published.[16] Analyses were performed by use of SPSS 28.

Funding and Contributions. No extramural funding was used to support this work. The authors are solely responsible for the design and conduct of this study, all study analyses, the drafting and editing of the paper and its final contents.

Results

Comorbidities. Of the 6,750 patients that had an ejection fraction of ≤50% and were included in this analysis, 33% had an ejection fraction of 36% to 50%. Seven percent of patients had ICDs at baseline and 12% prior to death. SCDs (n = 610 of 1892) made up 34% of all deaths in patients without an ICD (n = 572 of 1670) and decreased to 17% (n = 38 of 222) in patients with an ICD. The patients' baseline characteristics, **Table 1**, highlight that competing comorbidities were more prevalent in the non-SCD group. SCD was associated with higher systolic blood pressure and higher BMI. In contrast, non-SCD was associated with older age, higher prevalence of COPD and cancer, lower hemoglobin levels, worse kidney function, and increased diuretic requirements. A longer time until death was associated with a lower proportion of SCD (OR: 0.84/year, $P < 0.0001$). Expectedly, an ICD prior to death was associated with a lower proportion of SCD (OR: 0.38, $P < 0.0001$).

Discrimination. SHFM had good discrimination, with a 1-year area under the receiver-operating characteristic curve (AUC ROC) of 0.753, and good calibration, with a 5-year predicted mortality of 35.1% vs the observed 33.3%. SPRM was well-calibrated and discriminated SCD vs non-SCD in patients without an ICD and either EF ≤35% (OR: 1.96, $P < 0.0001$) or EF 36% to 50% (OR: 2.19, $P = 0.0002$), as well as a combination of both groups (OR: 2.06, $P < 0.0001$) (**Figure 1)**.

Predicted ICD benefit. Fifty percent of the trial participants in GISSI-HF had an EF ≤35% with a SPRM-estimated SCD risk ≥50%. The predicted ICD benefit in these patients was significant, as shown in **Figure 2**. Forty-three percent of the patients with an EF of 36% to 50% had an SPRM-estimated SCD risk ≥50%. The SPRM (OR: 1.92, $P < 0.0001$) accurately predicted the risk of

Journal Pre-proof

Journal Pre-proof

SCD vs non-SCD with an estimated proportion of SCD of 44% vs the observed proportion of 41% at 1 year. In order to understand the observed ICD benefit from SCD-HeFT to patients with EF ≤35%, we applied the ICD by SPRM interaction. Patients with SPRM ≥ vs <50% had a predicted all-cause mortality ICD benefit of 36% vs 4%. Similarly, among patients with an EF of 36% to 50%, patients with SPRM ≥ vs <50% had a predicted all-cause mortality ICD benefit of 35% vs 2%.

Regardless of whether the participants had an EF ≤35% or EF 36% to 50%, the 5-year mortality rates of patients with SPRM ≥50% show that SCD is similar to non-SCD in the first 2-year period (**Figure 3**). Patients with SPRM <50% had a higher proportion of non-SCD and are not anticipated to have a meaningful ICD benefit. 64.5% of the 6,750 patients met NYHA class II or III and EF ≤35% guideline criteria for a primary prevention ICD. These guideline criteria (EF ≤35%, NYHA class II or III) were not predictive of a higher proportion of SCD in patients without an ICD prior to death compared with those not guideline-indicated (35% vs 32%, OR: 1.16, P = 0.19). If an ICD were provided to these patients who are currently guideline indicated, the SPRM predicted benefit would be an estimated ICD HR of 0.81 for this cohort. Using SPRM >50% as an indication for an ICD, 47.8% may be eligible, including 30.2% with EF >35% (estimated ICD hazard ratio [HR] = 0.64). GISSI-HF participants with EF ≤35% with SPRM ≥50% had an estimated ICD HR of 0.64, comparable to patients with EF 36% to 50% with SPRM ≥50% (HR= 0.65), assuming the SPRM by ICD interaction remains similar in patients with EF 36-50%.

Journal Pre-proof

Additional Comorbidities. Among additional comorbidities added to the SPRM in a logistic regression model, clinical conditions such as ischemic etiology, stroke, peripheral artery disease, atrial fibrillation, QRS width, CABG/PCI, and guideline-directed medical therapy were not predictive of SCD vs non-SCD (**Table 2**). However, COPD (OR: 0.66, $P = 0.0002$) and a history of cancer (OR: 0.56, $P = 0.032$) were associated with a lower proportion of SCD, due to higher rates of non-SCD.

Discussion

We estimated the proportional risk of SCD, using the Seattle Proportional Risk Model, in 6,750 GISSI-HF trial participants who had an ejection fraction ≤50%. Two key findings were observed: (1) Only 50% of patients in GISSI-HF that meet current guidelines for ICD are predicted to benefit from ICD implantation; and (2) importantly, 43% of patients who do not meet current guidelines for ICD with an ejection fraction from 36% to 50% are predicted to benefit from ICD implantation.

Prior studies have demonstrated that the SPRM performs well to discriminate SCD compared with non-SCD and to predict variable ICD benefit, as shown in SCD-HeFT,[9] DANISH,[10] HF-ACTION,[11] and the NCDR ICD Registry.[12] In the prior analysis of SCD-HeFT, among patients with SPRM ≥50%, 5-year rates of SCD and non-SCD showed benefit from an ICD, with an observed 76% reduction in SCD and 44% reduction in all-cause mortality. The SCD rate was similar in those with SPRM <50% or ≥50% over 5 years, but the non-sudden death rate was twice as high in the latter follow up years with an overall ICD benefit of only 19% for SCD and a 10% non-significant excess for all-cause mortality. Similarly, in the current study in GISSI-HF

Journal Pre-proof

participants, the SCD rate was similar across EF values, but due to a higher non-SCD rate in the patients with SPRM predicted SCD <50%, the value of the ICD will be minimized.

Increased co-morbidities were associated with higher non-SCD mortality. An additional important component of this study was made possible by the GISSI-HF study design, which allowed us to test whether additional comorbidities altered the mode of death when added to the SPRM in a logistic regression model of SCD vs non-SCD. Among the variables studied, both COPD and a history of cancer increased the proportion of non-SCD, thus reducing the likelihood of patient benefit from an ICD. Both factors should be considered for addition to the SPRM. Ischemic etiology, QRS duration, guideline-directed medical therapy, and other comorbidities did not significantly alter the proportion of SCD in our analysis.

The results of predictive models, such as the SPRM, may demonstrate different outcomes based upon HF etiology. Overall mortality is well-recognized as being lower in NICM compared to those with ICM. For instance, in the DANSH trial, the only large randomized ICD trial since the SCD-HeFT study, no benefit of the ICD was observed over >5 years of follow up.[10] This trial enrolled 1,116 patients with nonischemic cardiomyopathy, left ventricular ejection fraction (LVEF) ≤35%, and NYHA II to III functional HF class to an ICD or no ICD. Patients were well treated with guideline-directed HF medications, and >50% of patients in both groups also received cardiac resynchronization therapy. However, even in this low-mortality patient population, the SPRM predicted a group of patients with a high proportional risk of SCD (median ≥54%) who had a substantial ICD benefit (n = 558, HR: 0.63, 95% CI: 0.43-0.94).[17]

Journal Pre-proof

SPRM is not the only risk prediction model developed to try to refine the target patient population for ICD therapy. A polygenic risk score utilized a genome-wide polygenic score for coronary artery disease for sudden and/or arrhythmic death risk stratification in an intermediate-risk population with established CAD but without severe systolic dysfunction, and found that patients in the top decile had a 77% increased risk of sudden and/or arrhythmic death.[18] In the DANISH-MRI study, the team sought to use late gadolinium enhancement on MRI to identify non-ischemic heart failure patients that would benefit from ICD implantation. Although late gadolinium enhancement did predict all-cause mortality in both ICD and control patients, it did not identify a group of patients that would survive longer after receiving an ICD.[19] CMR-GUIDE is currently testing ICD with presence of CMR-detected LGE in patients with an EF 36-50%.[20] REFINE-ICD is an ongoing randomized ICD study that utilizes abnormal heart rate turbulence[21] and T-wave alternans to risk stratify patients beyond 2 months post myocardial infarction with LVEF 36% to 50% (URL: https://www.clinicaltrials.gov. Unique identifier: NCT00673842). The PROFID consortium attempted to build a SCD risk model for ischemic post-myocardial infarction patients with EF 36-50% in over 200,000 patients and 85 variables, but were ultimately unsuccessful.[22]

Journal Pre-proof

Limitations

Limitations of this study include the post hoc nature of the present analyses, which were not pre-specified when GISSI-HF was designed. The risk prediction is based on clinical variables evaluated at baseline, and we cannot conclusively extrapolate our findings beyond the 4-year follow-up in GISSI-HF. Importantly, GISSI-HF occurred before more recent improvements in guideline directed medical therapy, and their contribution cannot be discounted. Further,

inclusion of study drug, even though randomized, could theoretically alter the population response. For these reasons, the findings from the current study should be considered hypothesis generating and in need of further validation.

Conclusions

The incremental value of this study is the prediction of patients who may benefit from a primary prevention ICD both by refining the subset of patients currently ICD-indicated to those who are more likely to benefit from an ICD and expanding the ICD indications to currently nonindicated patients (LVEF 36%-50%). Although the SPRM model is supportive of this approach to ICD selection, these results will need to be validated by a randomized controlled trial. Based on our work, using the SPRM multivariable risk model, a randomized trial enrolling heart failure patients with LVEF 36%-50%, SPRM ≥50% and a predicted SCD risk of ≥5% over 5 years would provide much needed data for understanding the risk of SCD and ICD benefit beyond LVEF ≤ 35%. It is essential that future primary prevention ICD studies are based upon patient populations selected according to their proportional SCD risk. Only in this way, can we protect patients from the risks of ICD implantation who will not benefit and similarly, provide life-saving ICD therapy to those with high SCD risk.

Journal Pre-proof

References

1. Yancy CW, Jessup M, Bozkurt B, Butler J, Casey DE, Jr., Colvin MM, Drazner MH, Filippatos GS, Fonarow GC, Givertz MM, Hollenberg SM, Lindenfeld J, Masoudi FA, McBride PE, Peterson PN, Stevenson LW and Westlake C. 2017 ACC/AHA/HFSA Focused Update of the 2013 ACCF/AHA Guideline for the Management of Heart Failure: A Report of the American College of Cardiology/American Heart Association Task Force on Clinical Practice Guidelines and the Heart Failure Society of America. *Circulation*. 2017;136:e137-e161.
2. McDonagh TA, Metra M, Adamo M, Gardner RS, Baumbach A, Bohm M, Burri H, Butler J, Celutkiene J, Chioncel O, Cleland JGF, Coats AJS, Crespo-Leiro MG, Farmakis D, Gilard M, Heymans S, Hoes AW, Jaarsma T, Jankowska EA, Lainscak M, Lam CSP, Lyon AR, McMurray JJV, Mebazaa A, Mindham R, Muneretto C, Francesco Piepoli M, Price S, Rosano GMC, Ruschitzka F, Kathrine Skibelund A and Group ESCSD. 2021 ESC Guidelines for the diagnosis and treatment of acute and chronic heart failure. *Eur Heart J*. 2021;42:3599-3726.
3. Al-Khatib SM, Stevenson WG, Ackerman MJ, Bryant WJ, Callans DJ, Curtis AB, Deal BJ, Dickfeld T, Field ME, Fonarow GC, Gillis AM, Granger CB, Hammill SC, Hlatky MA, Joglar JA, Kay GN, Matlock DD, Myerburg RJ and Page RL. 2017 AHA/ACC/HRS Guideline for Management of Patients With Ventricular Arrhythmias and the Prevention of Sudden Cardiac Death: Executive Summary: A Report of the American College of Cardiology/American Heart Association Task Force on Clinical Practice Guidelines and the Heart Rhythm Society. *Circulation*. 2018;138:e210-e271.
4. Bansch D, Antz M, Boczor S, Volkmer M, Tebbenjohanns J, Seidl K, Block M, Gietzen F, Berger J and Kuck KH. Primary prevention of sudden cardiac death in idiopathic dilated cardiomyopathy: the Cardiomyopathy Trial (CAT). *Circulation*. 2002;105:1453-8.
5. Kadish A, Dyer A, Daubert JP, Quigg R, Estes NA, Anderson KP, Calkins H, Hoch D, Goldberger J, Shalaby A, Sanders WE, Schaechter A, Levine JH and Defibrillators in Non-Ischemic Cardiomyopathy Treatment Evaluation I. Prophylactic defibrillator implantation in patients with nonischemic dilated cardiomyopathy. *N Engl J Med*. 2004;350:2151-8.
6. Strickberger SA, Hummel JD, Bartlett TG, Frumin HI, Schuger CD, Beau SL, Bitar C, Morady F and Investigators A. Amiodarone versus implantable cardioverter-defibrillator:randomized trial in patients with nonischemic dilated cardiomyopathy and asymptomatic nonsustained ventricular tachycardia--AMIOVIRT. *J Am Coll Cardiol*. 2003;41:1707-12.
7. Poole JE, Olshansky B, Mark DB, Anderson J, Johnson G, Hellkamp AS, Davidson-Ray L, Fishbein DP, Boineau RE, Anstrom KJ, Reinhall PG, Packer DL, Lee KL, Bardy GH and Investigators SC-H. Long-Term Outcomes of Implantable Cardioverter-Defibrillator Therapy in the SCD-HeFT. *J Am Coll Cardiol*. 2020;76:405-415.
8. Shadman R, Poole JE, Dardas TF, Mozaffarian D, Cleland JG, Swedberg K, Maggioni AP, Anand IS, Carson PE, Miller AB and Levy WC. A novel method to predict

the proportional risk of sudden cardiac death in heart failure: Derivation of the Seattle Proportional Risk Model. *Heart Rhythm*. 2015;12:2069-77.

9. Levy WC, Hellkamp AS, Mark DB, Poole JE, Shadman R, Dardas TF, Anderson J, Johnson G, Fishbein DP, Lee KL, Linker DT and Bardy GH. Improving the Use of Primary Prevention Implantable Cardioverter-Defibrillators Therapy With Validated Patient-Centric Risk Estimates. *JACC Clin Electrophysiol*. 2018;4:1089-1102.

10. Kristensen SL, Levy WC, Shadman R, Nielsen JC, Haarbo J, Videbaek L, Bruun NE, Eiskjaer H, Wiggers H, Brandes A, Thogersen AM, Hassager C, Svendsen JH, Hofsten DE, Torp-Pedersen C, Pehrson S, Signorovitch J, Kober L and Thune JJ. Risk Models for Prediction of Implantable Cardioverter-Defibrillator Benefit: Insights From the DANISH Trial. *JACC Heart Fail*. 2019;7:717-724.

11. Levy WC, Li Y, Reed SD, Zile MR, Shadman R, Dardas T, Whellan DJ, Schulman KA, Ellis SJ, Neilson M, O'Connor CM and Investigators H. Does the Implantable Cardioverter-Defibrillator Benefit Vary With the Estimated Proportional Risk of Sudden Death in Heart Failure Patients? *JACC Clin Electrophysiol*. 2017;3:291-298.

12. Bilchick KC, Wang Y, Cheng A, Curtis JP, Dharmarajan K, Stukenborg GJ, Shadman R, Anand I, Lund LH, Dahlstrom U, Sartipy U, Maggioni A, Swedberg K, O'Conner C and Levy WC. Seattle Heart Failure and Proportional Risk Models Predict Benefit From Implantable Cardioverter-Defibrillators. *J Am Coll Cardiol*. 2017;69:2606-2618.

13. Levy WC, Mozaffarian D, Linker DT, Sutradhar SC, Anker SD, Cropp AB, Anand I, Maggioni A, Burton P, Sullivan MD, Pitt B, Poole-Wilson PA, Mann DL and Packer M. The Seattle Heart Failure Model: prediction of survival in heart failure. *Circulation*. 2006;113:1424-33.

14. Tavazzi L, Maggioni AP, Marchioli R, Barlera S, Franzosi MG, Latini R, Lucci D, Nicolosi GL, Porcu M, Tognoni G and Gissi HFI. Effect of n-3 polyunsaturated fatty acids in patients with chronic heart failure (the GISSI-HF trial): a randomised, double-blind, placebo-controlled trial. *Lancet*. 2008;372:1223-30.

15. O'Connor CM, Whellan DJ, Lee KL, Keteyian SJ, Cooper LS, Ellis SJ, Leifer ES, Kraus WE, Kitzman DW, Blumenthal JA, Rendall DS, Miller NH, Fleg JL, Schulman KA, McKelvie RS, Zannad F, Pina IL and Investigators H-A. Efficacy and safety of exercise training in patients with chronic heart failure: HF-ACTION randomized controlled trial. *JAMA*. 2009;301:1439-50.

16. Chatterjee NA and Levy WC. Sudden cardiac death after myocardial infarction. *Eur J Heart Fail*. 2020;22:856-858.

17. Elming MB, Nielsen JC, Haarbo J, Videbaek L, Korup E, Signorovitch J, Olesen LL, Hildebrandt P, Steffensen FH, Bruun NE, Eiskjaer H, Brandes A, Thogersen AM, Gustafsson F, Egstrup K, Videbaek R, Hassager C, Svendsen JH, Hofsten DE, Torp-Pedersen C, Pehrson S, Kober L and Thune JJ. Age and Outcomes of Primary Prevention Implantable Cardioverter-Defibrillators in Patients With Nonischemic Systolic Heart Failure. *Circulation*. 2017;136:1772-1780.

18. Sandhu RK, Dron JS, Liu Y, Moorthy MV, Chatterjee NA, Ellinor PT, Chasman DI, Cook NR, Khera AV and Albert CM. Polygenic Risk Score Predicts Sudden Death in Patients With Coronary Disease and Preserved Systolic Function. *J Am Coll Cardiol*. 2022;80:873-883.

Journal Pre-proof

19. Elming MB, Hammer-Hansen S, Voges I, Nyktari E, Raja AA, Svendsen JH, Pehrson S, Signorovitch J, Kober L, Prasad SK and Thune JJ. Myocardial fibrosis and the effect of primary prophylactic defibrillator implantation in patients with non-ischemic systolic heart failure-DANISH-MRI. *Am Heart J*. 2020;221:165-176.
20. Selvanayagam JB, Hartshorne T, Billot L, Grover S, Hillis GS, Jung W, Krum H, Prasad S and McGavigan AD. Cardiovascular magnetic resonance-GUIDEd management of mild to moderate left ventricular systolic dysfunction (CMR GUIDE): Study protocol for a randomized controlled trial. *Ann Noninvasive Electrocardiol*. 2017;22.
21. Disertori M, Mase M, Rigoni M, Nollo G and Ravelli F. Heart Rate Turbulence Is a Powerful Predictor of Cardiac Death and Ventricular Arrhythmias in Postmyocardial Infarction and Heart Failure Patients: A Systematic Review and Meta-Analysis. *Circ Arrhythm Electrophysiol*. 2016;9.
22. Dagres N, Peek N, Leclercq C and Hindricks G. The PROFID project. *Eur Heart J*. 2020;41:3781-3782.

Journal Pre-proof

Table 1 Baseline Characteristics of GISSI-HF Patients with EF ≤50%

	Total	Alive	SCD	nonSCD	SCD vs. nonSCD *P*-value
N	6750	4858	610	1282	
Age	67 (11)	65 (11)	70 (9.2)	72 (9)	<0.001
Male (%)	79%	78%	83%	82%	0.50
NYHA Class	2.4 (0.5)	2.3 (0.5)	2.5 (0.6)	2.6 (0.6)	0.004
Ejection Fraction (%)	32 (7)	33 (7)	31 (7)	31 (8)	0.54
SBP (mmHg)	126 (18)	127 (18)	126 (18)	123 (18)	0.007
Ischemic etiology	50%	47%	60%	49%	0.58
BMI	27.0 (4.4)	27.3 (4.4)	27.0 (4.3)	26.0 (4.3)	<0.001
Previous CABG	19%	17	21%	24%	0.23
Previous PCI	13%	13	11%	35%	0.90
Diabetes Mellitus	28%	26%	32%	24%	0.15
History of Atrial Fibrillation	19%	17%	23%	11%	0.63
History of CVA	5%	4%	5%	7%	0.10
History of PAD	9%%	7%	12%	14%	0.15
History of COPD	22%	18%	26%	33%	0.001
History of Cancer	4%	3%	3%	5%	0.029
QRS Duration (ms)	119 (35)	116 (34)	124 (35)	126 (35)	0.26
ACEi Use	77%	79%	76%	73%	0.12
ARB Use	19%	18%	19%	21%	0.30
Beta Blocker Use	66%	70%	58%	54%	0.12
MRA Use	39%	38%	41%	44%	0.22
Baseline Statin Use	23%	24%	22%	21%	0.22
Digoxin Use	37%	33%	46%	47%	0.60
Allopurinol Use	21%%	17%	31%	31%	0.83
ICD Baseline	7%	7%%	5%	11%	<0.001
ICD Prior to Death	12%	12%	6%	14%	<0.001
Furosemide (mg/kg/day)	0.81 (0.94)	0.66 (0.69)	1.03 (1.02)	1.28 (1.43)	<0.001
Sodium	140.0 (3.8)	140.1 (3.7)	139.8 (4.0)	139.8 (3.8)	0.69
Creatinine	1.20 (0.49)	1.14 (0.43)	1.32 (0.55)	1.39 (0.6)	0.007
Uric Acid	6.7 (2.0)	6.5 (1.9)	7.0 (2.1)	7.1 (2.2)	0.28
WBC	7.4 (2.1	7.3 (2.1)	7.3 (1.9)	7.5 (2.2)	0.26
Lymphocytes (%)	20.9 (2.4)	21.2 (2.3)	20.5 (2.5)	19.9 (2.4)	
Hemoglobin	13.7 (1.6)	13.9 (1.6)	13.5 (1.8)	13.3 (1.7)	0.009
Total Cholesterol	191 (43)	193 (42)	188 (46)	183 (44)	0.24
SPRM (%)	48.9 (11.9)	50.9 (11.4)	46.3 (11.4)	42.7 (11.4)	<0.001
SPRM >50%	47.8%	54.5%	37.4%	27.0%	
SHFM 1 yr survival	0.914 (.088)	0.932 (0.060)	0.883 (0.109)	.861 (.130)	0.022
SHFM 5 yr Survival	0.709 (0.192)	0.751 (0.157)	0.633 (0.222)	.585 (0.23)	0.047

Data are displayed as mean (standard deviation). ICD placement refers to placement by end of study or patient death.

[a]Abbreviations: ACEi = angiotensin-converting enzyme inhibitor; ARB = angiotensin receptor blocker; BMI = body mass index; COPD = chronic obstructive pulmonary disease; CVA = cerebrovascular accident; ICD = implantable cardioverter-defibrillator; MRA = mineralocorticoid receptor antagonist; NYHA = New York Heart Association; PAD = peripheral artery disease; SCD = sudden cardiac death; SHFM = Seattle Heart Failure Model; SPRM = Seattle Proportional Risk Model; WBC = white blood count.

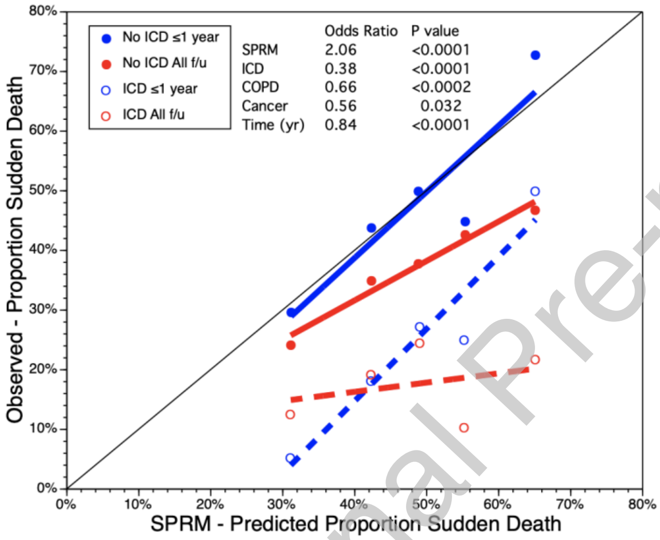

Figure 1 Calibration of the SPRM in GISSI-HF patients. The SPRM-predicted proportion of SCD is compared with the observed proportion of SCD. f/u = follow-up; other abbreviations as in Table 1.

Journal Pre-proof

A

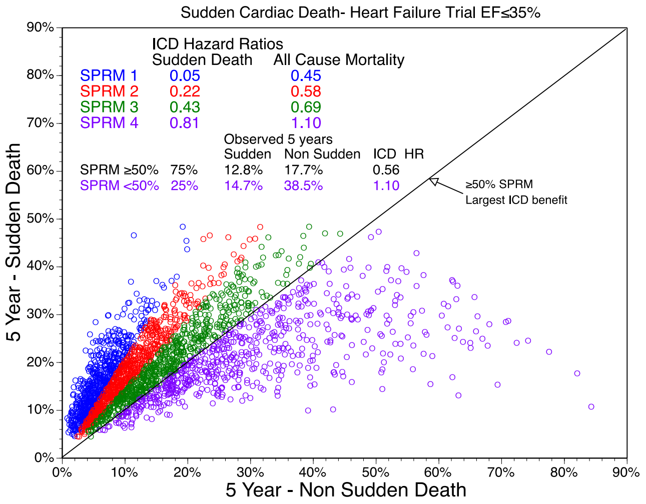

B

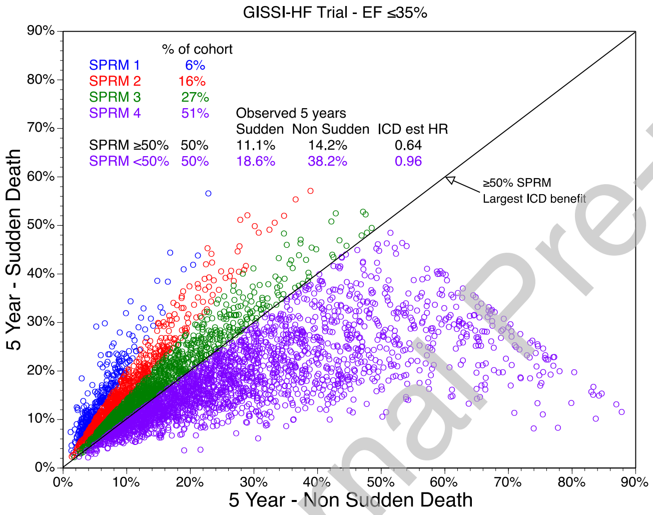

C

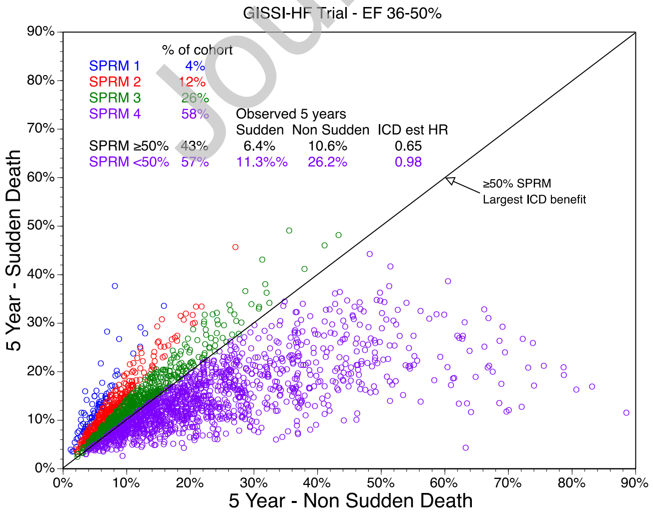

Journal Pre-proof

Figure 2 The estimated 5-year SCD rate vs. the 5-year non-SCD rate by SPRM are shown for SCD-HeFT and GISSI-HF (EF ≤35% and EF 36-50%). The quartile cutoffs of the SPRM within SCD-HeFT and the corresponding observed ICD hazard ratio is shown for Sudden Death and All-Cause Mortality. Patients within each color SPRM quartile have similar observed ICD benefit despite varying rates of SCD in SCD-HEFT. Within the GISSI-HF cohort, the % of the cohort that is within the corresponding SPRM quartile, using the SCD-HeFT quartile cutoffs is shown. The observed 5 year sudden and non-sudden death rates are shown for SPRM ≥ and <50% with the estimated ICD benefit based on the SPRM-ICD interaction observed within SCD-HeFT and programmed in the online interactive SPRM model. (A) SCD-HeFT analysis, EF ≤35%; (B) GISSI-HF analysis, EF ≤35%; (C) GISSI-HF analysis, EF 36% to 50%. Abbreviations as in Table 1.

A

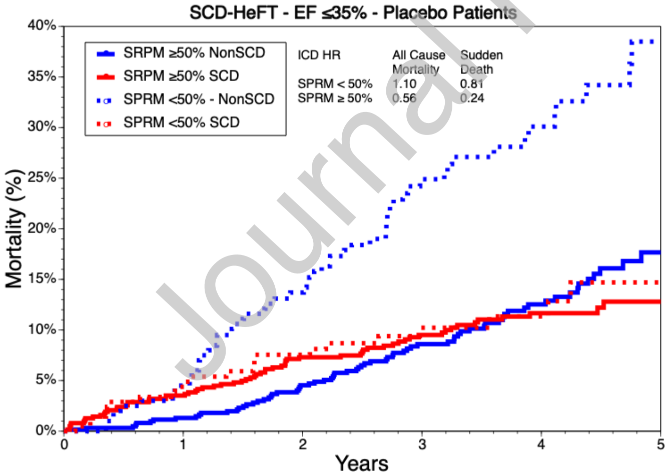

B

Journal Pre-proof

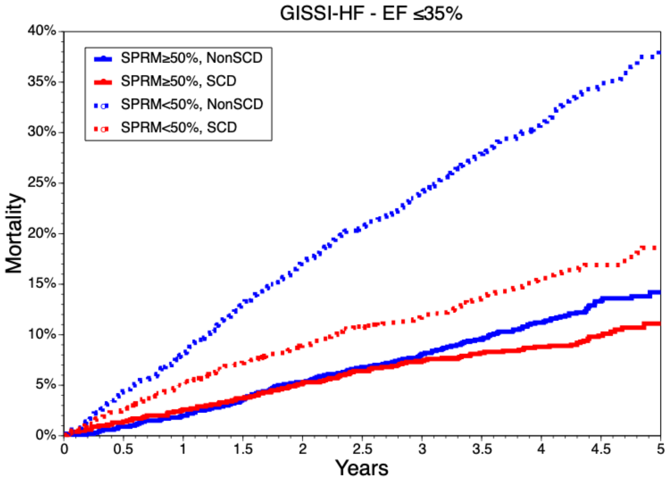

C

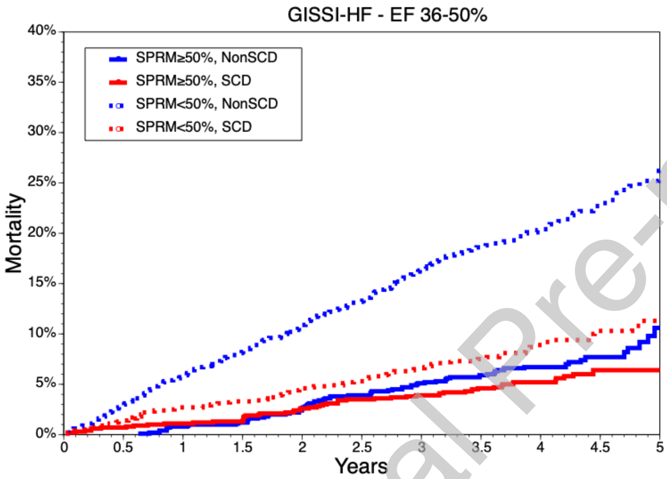

Figure 3. Kaplan-Meier curves showing rates of SCD vs non-SCD risk stratified by SPRM <50% or ≥50% and EF for (A) SCD-HeFT, and GISSI-HF with (B) EF ≤35% and (C) EF 36% to 50%. Abbreviations as in Table 1.

Journal Pre-proof

Table 2 Multinomial Logistic Regression odds ratios for SCD vs non-SCD

	Univariable Odds Ratio	95% CI		*P*-value	Multivariable Odds Ratio	95% CI		*P*-value
**SPRM Score**	1.83	1.51	2.22	<0.001	2.01	1.64	2.46	<0.001
**SHFM Score**	0.756	0.67	0.85	<0.001				
**ICD Prior to Death**	0.396	0.28	0.57	<0.001	0.376	0.26	0.54	<0.001
**Years to Death**	0.876	0.81	0.94	<0.001	0.844	0.78	0.91	<0.001
**Ischemic Etiology**	1.06	0.87	1.29	0.58				
**History of CVA**	0.71	0.47	1.07	0.10				
**CABG or PCI**	0.91	0.74	1.13	0.39				
**History of Atrial Fibrillation**	0.94	0.75	1.19	0.63				
**History of PAD**	0.81	0.6	1.08	0.15				
**History of COPD**	0.71	0.57	0.88	0.002	0.70	0.57	0.88	0.002
**History of Cancer**	0.57	0.34	0.95	0.031	0.59	0.35	0.99	0.047
**QRS Duration (10ms)**	0.98	0.96	1.01	0.26				
**ACEi Use**	1.19	0.95	1.49	0.12				
**ARB Use**	0.88	0.69	1.12	0.30				
**Beta Blocker Use**	1.17	0.96	1.42	0.12				
**MRA Use**	0.88	0.73	1.08	0.22				

[a]Abbreviations: CABG = coronary artery bypass grafting; PCI = percutaneous coronary intervention; other abbreviations as in Table 1.

Journal Pre-proof
